# Rectus Sheath Hematoma

**DOI:** 10.7759/cureus.61488

**Published:** 2024-06-01

**Authors:** Jai Gupta, Sana Tahir, Amelie Oshikoya, Latha Ganti

**Affiliations:** 1 Biology, Seminole Science, Lake Mary, USA; 2 Medicine, Orlando College of Osteopathic Medicine, Winter Garden, USA; 3 Research, Orlando College of Osteopathic Medicine, Winter Garden, USA; 4 Medical Sciences, The Warren Alpert Medical School of Brown University, Providence, USA; 5 Emergency Medicine and Neurology, University of Central Florida College of Medicine, Orlando, USA

**Keywords:** lower abdominal pain, rectus abdominis (ra), rare cause of acute abdominal pain, rectus sheath hematoma, spontaneous rectus sheath hematoma

## Abstract

The authors report on the case of a 69-year-old female who presented to the emergency department due to exquisite abdominal pain she described as occurring after she coughed. Imaging revealed a rectus sheath hematoma (RSH). A RSH is an uncommon but significant cause of acute abdominal pain that occurs when blood accumulates in the sheath of the rectus abdominis muscle. It can be caused by a muscular tear or a ruptured epigastric artery and can happen spontaneously or after trauma. The etiology, presentation, diagnosis, and management are discussed.

## Introduction

A rectus sheath hematoma (RSH) occurs when blood accumulates in the rectus sheath muscle in the abdomen. Bleeding into the rectus sheath occurs after damage to the epigastric arteries or by direct muscle tear [1]. The rectus sheath is a fibrous compartment encasing the rectus abdominis muscle. Bleeding from the inferior or superior epigastric artery is the most common cause of the condition. RSH can occur in various locations and be of different sizes [2]. A forceful contraction of the rectus abdominis can cause the injury [1].

In some cases, RSH can have a complication called abdominal compartment syndrome, which requires endovascular and surgical intervention. The use of antiplatelets or anticoagulants increases the risk of an RSH. RSH's overall mortality rate can be estimated at around 4%; however, with the use of anticoagulation, this increases to 25% in patients [1]. A higher incidence of RSH also increases the number of further complications, such as abdominal compartment syndrome. RSH is less common in men when compared to women, as they lack muscle mass in comparison. Pregnancy can be a predisposing factor to the development of RSH [1], as can COVID-19 [3].

Diagnosing RSH can be difficult due to its symptoms mirroring a number of intra-abdominal pathologies [4]. RSH accounts for only 2% of patients who present with acute abdominal pain; however, if left untreated, it can develop into requiring a laparotomy or even death [1]. Most patients complain of abdominal pain and abdominal wall mass as the primary presenting symptom [5]. Abdomen and pelvis CT scans are the most common method to diagnose RSH in patients. Blood transfusion and symptom management are the most common methods of treatment for patients. However, some require surgical intervention or endovascular embolization of bleeding vessels [5]. CT of the abdomen and pelvis, along with physical examination and past medical history collection, can aid in rapid diagnosis, decreasing fatal outcomes for patients presenting with RSH [5].

## Case presentation

A 69-year-old female patient presented to the emergency department with left-sided lower abdominal pain for one day. She says it felt like a ball in her lower abdomen. She denied any trauma. She said it occurred after a particularly forceful episode of coughing, which she attributed to her usual smoking.

She had no fevers, chills, chest pain, shortness of breath different from her baseline, nausea, vomiting, diarrhea, headache, or focal weakness. Her medical history was significant for chronic obstructive pulmonary disorder (COPD), for which she was on albuterol. She was taken off her hyperlipidemia and hypertension medication two years prior. Her vital signs were significant for hypertension, with a blood pressure of 180/102 mmHg and mild room-air hypoxia with an oxygen saturation of 92% (consistent with her known COPD).

Laboratory analysis was significant for mild hyponatremia, hypochloremia, and thrombocytopenia (Table 1).

**Table 1:** Patient's laboratory results

Test	Value	Reference range
Chemistry		
Sodium	128	135–145 mmol/L
Potassium	3.9	3.5–5.3 mmol/L
Chloride	91	99–111 mmol/L
Carbon dioxide	29	21–32 mmol/L
Blood urea nitrogen	5	7–22 mg/dL
Creatinine	0.5	0.6–1.3 mg/dL
Glucose	138	74–106 mg/dL
Calcium	8.8	8.4–10.2 mg/dL
Total bilirubin	0.4	0.0–1.0 mg/dL
Aspartate aminotransferase	34	7–37 Units/L
Alanine aminotransferase	25	12–78 Units/L
Total alkaline phosphatase	92	50–136 Units/L
Total Protein	7.9	6.4–8.2 g/dL
Albumin	4.2	3.4–5.0 g/dL
Hematology		
White blood cell count	7.3	4.0–10.5 × 10^3^/μL
Red blood cell count	5.03	3.93–5.22 × 10^6^/μL
Hemoglobin	16	11.2–15.7 × g/dL
Hematocrit	46	34.1–44.9%
Mean corpuscular volume	91.5	79.4–94.8 fL
Mean corpuscular hemoglobin	31.8	25.6–32.2 pg
Red cell distribution width	13.4	11.7–14.4%
Platelet count	128	150–400 × 10^3^/μL

On a focused physical examination, the patient appeared well and showed no signs of acute distress. She was awake and alert and exhibited a normal mental status. The patient's abdomen was atraumatic and non-tender to palpation. There were no signs of guarding or rebound tenderness, and bowel sounds were within the normal range. No abdominal distention or palpable mass was observed, and there was no evidence of a pulsatile mass. The musculoskeletal examination of the back showed no abnormalities. The patient exhibited a full range of painless motion, and there was no tenderness along the midline vertebral area or paraspinal region. Muscle spasm was not present, and the straight leg raise test was negative, indicating no signs of sciatica or nerve compression. Moreover, there was no tenderness in the costovertebral angle.

**Figure 1:**
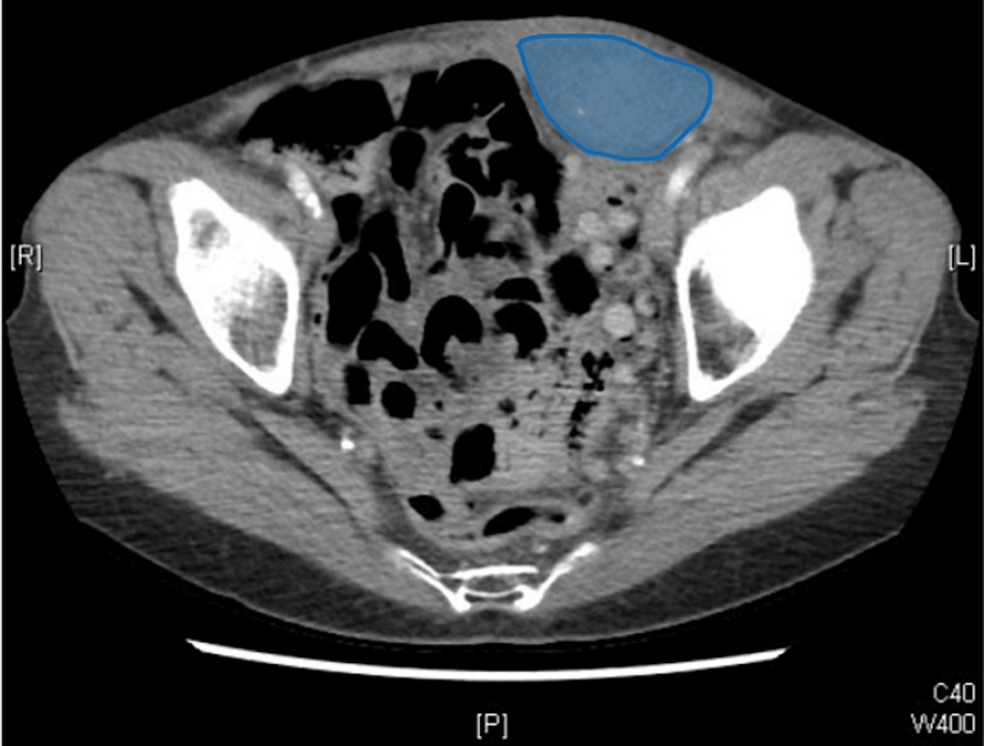
Computed tomography scan demonstrating a large left rectus sheath hematoma measuring approximately 15.0 cm × 4.4 cm × 5.8 cm

A CT scan of the abdomen and pelvis demonstrated a large left rectus sheath hematoma measuring approximately 15.0 cm × 4.4 cm × 5.8 cm (Figure 1). 

General surgery was consulted, and given that the patient was stable, they elected to admit the patient for observation rather than immediate surgical drainage of the hematoma. Throughout the hospitalization, the patient's hemoglobin levels remained stable, and a follow-up CT scan on day 3 showed no signs of active extravasation.

To explore the reasons for her cough, a viral panel was conducted, but no specific upper respiratory infection was identified. The patient mentioned cleaning her house every day using chloride and other household products in non-ventilated areas. She was advised to use such products only in ventilated areas to minimize the worsening of her shortness of breath and cough.

In assessing her functional status, a six-minute walking test was performed, and it was found that the patient did not require home oxygen. Consequently, she was discharged in fair condition with appropriate recommendations.

## Discussion

Rectus sheath hematomas are rare clinical events. They should be considered when looking at a patient with pain in the abdomen. Major risk factors include anticoagulants and blood thinners. Anticoagulant medication increases the risk of bleeding. However, these medications do not cause bleeding directly but can aggravate an existing hemorrhagic episode [6]. Other risk factors include pregnancy, obesity, and trauma. These risk factors all target the epigastric artery and its branches. These risk factors all increase stress on the epigastric artery. An observational study of 61 patients showed that 91.8% (56) suffered from at least one chronic disease, whereas 77% were currently on anticoagulation therapy [6]. It was determined that patients who took acetylsalicylic acid had larger RSH sizes when compared to those who took other anticoagulants [7].

Large rectus sheath hematomas usually occur under the arcuate line because of the absence of the posterior rectus sheath. Hematomas occurring below the arcuate line occur from bleeding in the inferior epigastric artery that spreads extraperitoneally if the peritoneum is ruptured. Hematomas below the arcuate line typically lose more blood before hematoma detection. Hematomas that occur below the arcuate line are most commonly caused by bleeding of the inferior epigastric vessels [8]. Therefore, they can dissect into the retroperitoneal space, allowing for longer detection times and increased quality of blood loss. Hemorrhage within this area minimizes any natural tamponade effect [8].

The main features of RSH are pain in the abdomen, usually with mass. There are also several other symptoms, such as fever, weakness, and confusion. Patients can report symptoms of nausea, vomiting, syncope, and urinary retention [9]. Appendicitis, diverticulitis, colitis, or ovarian abscess can be considered prior to RSH due to the non-specific symptom presentation of RSH [9]. With the use of radiological imaging, such as CT imaging with IV contrast, an accurate diagnosis of RSH can be completed. Abdominal bruising typically occurs later.

Treatment is usually conservative if there are no complications. RSH complications are rare but can lead to abdominal compartment syndrome, infection, or myonecrosis in some cases. Conservative management usually includes fluid resuscitation, reversal of anticoagulants, lab monitoring, and bed rest. Some patients require quick intervention to embolize an actively bleeding artery. Regardless of conservative or rapid treatment, patients are advised to halt the use of any anticoagulation medication to avoid exacerbating RSH symptoms [9].

## Conclusions

Rectus sheath hematoma is a relatively rare presentation that can occur after a seemingly benign trigger, such as coughing. It can be managed conservatively, as in this case, or if it does not resolve spontaneously, it will occasionally require operative repair.
